# "The Hospital" Nursing Mirror

**Published:** 1897-07-31

**Authors:** 


### "The Hospital" Nursing Mirror.

Being the Nursing Section of "The Hospital."

[Contributions for this Section of "The Hospital" should be addressed to the Editor, The Hospital, 28 & 29, Southampton Street, Strand, London, W.C., and should have the word "Nursing" plainly written in left-hand top corner of the envelope.]

### News from the Nursing World.

### A SOCIAL GATHERING.

The social meeting of the Royal British Nurses' Association was very successful. Dr. Langton, the hon. treasurer of the association, and Mrs. Langton held a reception at 62, Harley Street, at which H.R.H. Princess Christian, accompanied by the Baroness Von and Zu Egloffstein, and 400 guests were present. Several nurses from the Colonies were amongst them, and were presented to the Princess, at her request. The following ladies and gentlemen were also there: Lily, Duchess of Marlborough, Lord William Beresford, Lady Jane Taylor, Sir Frank and Lady Lockwood, Sir Borrodaile and Lady Savory, Sir Dyce and Lady Duckworth, Lady Priestley, Sir James Crichton Browne and Lady Browne, Sir Richard and Lady Thorne, Sir Henry Burdett, Sir W. and Lady Agnes Cooper, Professor and Mrs. Max Müller, the Earl and Countess of Lindsay, Sir E. and Lady Saunders, Sir Thomas Smith, &c.

### THE BABIES AT THE VICTORIAN ERA EXHIBITION.

The babies in process of incubation at Earl's Court are in the care of French nurses, of whom there appear to be three. One is a wet nurse, mother's milk being, so far, superior to any substitute. The manner of dressing the children is not in accordance with Jaeger-trained minds, for the principal part of the clothing is of stiff cotton damask. The outside raiment is in two pieces, a little jacket with long sleeves and a breadth of the same stuff wrapped round the legs and folded up behind neat and square, so that the lower portion of the baby has the appearance of a nice white pillow, whilst the upper part allows the hands and head to be seen, neither lovely at this state of existence. In the nursery a padded table is provided, upon which the child is laid whilst its toilet is performed, and deftly performed, by the nurse. Beside it is a pair of scales (a pink-lined basket on one side, and the weights on the other), and every baby is weighed before and after a meal, and a record kept of the amount of food taken.

### HEYWOOD DISTRICT NURSING ASSOCIATION.

The annual report of the Heywood District Nursing Association shows a successful year's work, and the support of an increasing number of donors and subscribers. Eight pounds eighteen shillings has also been received from paying patients as thank-offerings; and this the chairman, Alderman Healey, rightly regarded as a most satisfactory item. The scheme to commemorate the year by the erection of a cottage hospital or nurses' home has not been lost sight of, and it is hoped will some day be undertaken.

### INQUEST AT NORTH DUBLIN UNION.

Had any example of the evils of pauper nursing been needed, the inquest on an infant at the North Dublin Union would have supplied it. The Local Government Board may exercise a paternal supervision over the diet of unfortunate children, but when we learn that that appointed for an infant under 12 months is "2 pints milk, ½-pint beef tea, 1 oz. sugar, 6 oz. bread, and 2 oz. rice," the need of a trained woman is shown to be urgently necessary. The verdict of mans'aughter is only the natural result of starving children to death by giving them improper food, and th s child's martyrdom—and alas! it is only one of many—will not have been in vain if it helps to sweep away the pauper nursing still so common in the workhouses of the United Kingdom.

### THE MACCLESFIELD NURSING SCHEME.

Much more must be known of the Macclesfield nursing scheme before the satisfaction with which it was announced can be justified. The committee of the Board of Guardians have declared themselves ready to increase their infirmary staff by two nurses of not less than two years' training, and say that the services of any two nurses on the staff who have the qualification shall be available for private work to those who can afford to pay one or two guineas a week (according to the nature of the illness). When not engaged in private work the nurses will be on duty in the infirmary wards, and will receive the munificent salary of £20 per annum for the first year, £22 10s. the second, and £25 the third. In spite of the profit that must arise by hiring nurses at £25 per annum and letting them out at from £50 to £100 per annum and getting a fair share of work out of them in the infirmary wards, the public are invited to support the scheme liberally. The salaries of the nurses should not be taxed to yield a profit to the infirmary. Nor do we see why the public should subscribe to support an institution that, if well managed, should be a commercially profitable undertaking.

### SHEFFIELD NURSES' HOME.

The twenty-sixth annual report shows a successful year's working. The earnings of the nurses have been £1,616, the subscriptions £147, the expenditure £1,578. The balance has been carried forward to the capital account. Mrs. Overend has made a gift of £1,000 to the superannuation fund, which now stands at £1,169.

### SUFFERING AT INNISKEA.

Mrs. Kildare Treacy, of the City of Dublin Nursing Institution, is appealing for help for the fever-stricken island. The nurses on duty report a state of appalling poverty. "I never thought there was such poverty in the world until I came here. . . . There are pigs, cows, or donkeys in nearly every house." One of the two nurses originally sent has taken the fever, and there are 20 patients seriously affected. Two more nurses are asked for. The nurse's remark about the poverty hardly seems carried out by the presence of "pigs, or cows, or donkeys," for these animals represent the wealth of the poor. Dirt, rags, hovels, and mismanagement there are doubtless, and the need for immediate help is urgent; but any highhanded action of the Government to institute a better order of things would probably be strongly resented by the islanders.

### THE NURSE'S HOLIDAY.

In honour of the Queen's record reign, the servants and matron of the Cardiff Infirmary have been granted an extension of holiday leave. This privilege has not been accorded to the nurses, and accordingly 21 nurses, of whom 12 were probationers, signed a letter to the committee asking for a week's extra leave and one day off a month. This letter is under consideration, and is the cause of a considerable "rift within the lute" in the committee's councils, the chairman being of opinion that the matron's authority must be upheld, whilst Dr. C. Vachell considered that there were signs of disquietude that ought to be investigated. The matter was referred to the Nursing Committee.

### LIVERPOOL DISTRICT NURSING SCHEME.

The nursing scheme by which Liverpool celebrates the Record Reign is simple and comprehensive. Mr. William Rathbone, who promoted the idea, was much disappointed by the laggardly way in which the subscriptions to the last fifth of the necessary £30,000 were coming in, but all anxiety has been removed by a communication he has lately received from the David Lewis Trustees, offering to build the central home and office in the city; and, if the committee submit a scheme meeting with their approval, possibly the other two homes which may be required. The news has given great satisfaction, and in providing trained nursing for the city the trustees certainly fulfil, in letter and spirit, the desire of the late Mr. David Lewis, who left the magnificent sum of £350,000 in their hands for the benefit of the poor in Liverpool and Manchester.

### GARDEN PARTY AT MOSELEY COLLEGE, BIRMINGHAM.

The Moseley Botanical Gardens were lent to the Sparkhill, Greet, and District Nursing Guild for the purpose of raising funds to provide a nurse. Various entertainments—concerts, aquatic displays, and an athletic tournament—were given, and the members of the guild took advantage of the occasion to present Mrs. Sam Mason, one of the vice-presidents, with an illuminated address, a lady's hand-bag, and a handsome purse, in recognition of her services and in token of their esteem. Mrs. Green, an active member of the committee, was also presented with a handsome gold brooch.

### GOOD WORK IN WALES.

Miss S. Peter, St. Katherine's Royal Hospital, after inspecting the Trevor, Garth, and Vroncysyllte Nursing Association, reports approvingly of the work done. The nurses' work is good, equipment neat, and the books carefully kept, whilst the nurse is popular, and there is a balance to the good of £24. This result is owing in a great measure to the energy with which the secretary and treasurer, Mrs. E. Lloyd Edwards, discharged her duties. She was unanimously reappointed to these offices. Some changes are contemplated in the number of the committee, and the question has been raised of admitting the neighbouring districts of Pontcysyllte and Trefynant to share in the work of the association.

### FOR SICK NURSES.

At the convalescent home recently opened by the Princess Louise for the patients of the Metropolitan Hospital two charming bed-rooms are provided for sick nurses belonging to the hospital staff. They occupy corners close by the day-rooms, and are furnished well and tastefully. The walls are distempered in the same colours as the rest of the home. A large square window juts out at the corner, and a tiny tiled hearth gives the promise of a cosy corner in chilly weather. The bed is of the usual pattern, with hair mattress, and a substantial occasional table, large enough to write at, yet small enough for afternoon tea, can be moved about at will. The rest of the furniture consists of a dressing-chest, a wardrobe, with long glass, &c., of light polished wood. The floor is covered with cork carpet, and beside the bed a bright-coloured oriental mat is spread. So far as situation and comfort is concerned it is difficult to imagine a more ideal retreat in which to gain strength after sickness.

### EAST END NURSING.

Lady Frederick Cavendish, Lady Florence Cecil, and Mrs. Robert Franks are appealing for funds to carry on the Haggerston and Hoxton District Nursing Association. The need is urgent, as unless help be forthcoming the association must part with one of the nurses, and this with a record of 25,000 visits paid by the nurses during the last twelve months, and in face of a still greater demand for their services. The funds are economically managed; seven nurses are maintained, and the income for the year has not been quite £600. The Treasurer, 80, Nichols Square, Hackney Road, N.E., will be grateful for subscriptions or donations.

### SHORT ITEMS.

The nursing staff of Ennis Workhouse Infirmary consists of three nuns, a night nurse, a day nurse, wards' maid and wards' man, and is in charge of 200 sick persons, of whom 46 are lunatics. The Government local inspector does not think it large enough, and the Guardians are in a rebellious frame of mind.—The Medical Inspector, criticising the nursing arrangement at the request of the Guardians of Yarmouth, said "the present system of dual control was bad, and did not work well. The ideal was to have a matron trained nurse, who would be the superintendent over two charge nurses, the one for the male and the other for the female wards.—At Llanrwst a Welsh-speaking nurse is to be appointed as parish nurse.—A cheque of £408, the proceeds of the St. Vincent Ball on behalf of the St. Vincent's Hospital, Sydney, was publicly presented by Lady Hampden to the Sister Superior on behalf of the hospital on the last day of April.—The plans of Mr. Henry Lord have been selected by the General Arrangement Committee for the Salford Nurses' Home, which is to be built in honour of the Queen's long reign. — After the examination on "General Physiology, and Matters Pertaining to Nursing," held by Dr. Fletcher, Resident Physician of the Aberdeen City Hospital, at the close of his lectures the committee decided to present prize volumes to Nurses Taylor, Margaret Clark, Law, and Mary Duncan.—Only £100 is now needed to start the "Victoria Nurses' Home" for Chesterfield.

### Lectures to Surgical Nurses.

By H. A. LATIMER, M.D. (Dunelm), M.R.C.S. of Eng., L.S.A. of London, Consulting Surgeon, Swansea Hospital; President of the Swansea Medical Society; Lecturer and Examiner of the St. John Ambulance Association, &c.

(*Continued from page* 139.)

### IX.—INFLAMMATION.

IF we go further, now, and look to the causes of inflammation, we shall do well to divide that matter up into the two headings of constitutional and local irritations. As these are lectures on surgical nursing, I do not think it necessary to say more on the first point than that a variety of irritations acting within the body may set up the process. If a person is suffering from diseases which, by interfering with his being able to get rid of poisons formed within his system, result in his blood being loaded with faulty and irritating materials, inflammation will be excited in the body. In this way gout and diabetes act in causing inflammation. And if poisons, such as lead and alcohol, have gained an admission to the body, they will also irritate and inflame the tissues. Starvation and anæmia (or poorness of the blood, itself), by inducing faulty action of organs, or by starving them of their proper supply of stimulation and nourishment, will act in the same way. It will suffice, now, to note these facts, and we will pass on to the question in its surgical aspect.

The great causes of surgical inflammations, then, are injuries of various kinds. There need not necessarily be a wound to account for an inflammation; an injury may have disturbed the health or vitality of the part without leading to any breakage of the skin, and yet serious local disturbance of function may result. A man, say, has sprained his ankle, and this sprain is quickly followed by the redness, heat, pain, and swelling which mark the inflammatory state; and any of the results of that condition, which I shall have to tell you of shortly, may follow later on. Or a simple fracture of a bone may, under unfavourable circumstances, be the prelude to an inflammation at the seat of breakage. It is not at all difficult to see how the mischief is set up in such cases; for, under the irritation to which the injured parts are subjected by the force exercised by the blow or strain which has affected them, blood-vessels are broken, nerves are crushed or torn, and other structures are in various ways injured. All processes of life in the body are controlled by the nervous system, and if any of the nerves which are carrying nerve force are interfered with prejudiciously, disturbance of life in the parts controlled by those nerves must result. I will here give you two familiar examples of the control which is exercised over blood-vessels by nerves. You all know, when a sudden fright alarms a person, how white his face is apt to become, and you remember that he is then said to have become "blanched with fear." And, again, the more common-day occurrence of blushing must be one which has been noticed by you—a condition in which the face has become red and hot under some passing emotion. In both these cases the changes in colour have resulted from orders telegraphed to nerves of the so-called sympathetic nervous system from the brain. All blood-vessels have little threads of those nerves distributed in their walls, and in response to irritations conveyed by these nerves the blood-vessels enlarge or contract in size, more or less blood is contained in them, and the outward change of colour of blushing or pallor results. Under certain circumstances of disturbed nerve force the process goes a step further than mere curtailment or excess of blood in a part, and then inflammation steps in.

But inflammations of the sort we have been considering are mostly kindly and beneficial in their results, and it is not until we have a new agent making its appearance upon the scene that there are evil consequences to them. This new agent is supplied by a germ. There are an infinite number of germs, each capable of setting up its own peculiar or specific variety of disease when it has gained access to the body. I have already defined a germ as a seed-bud. Another name by which a germ has become to be known by us is a "micro-organism"—that is, a structure which is of so minute a size that it can only be seen by a microscope. There is no doubt now as to the part played by germs in causing disease. There was a time, not so very many years ago too, when certainty did not exist as to the ravages they effected; but now, thanks largely to the labours of Lister and Pasteur, the circumstances under which they exist, the means by which they gain access to the body, the modes by which they propagate themselves and do their mischief, and the means by which they can be recognised, hindered in their deadly work, or killed, are largely known. Nothing is more sure now in science than the fact that, under proper precautions, wounds can be intententionally made whilst the germs which ordinarily affect those wounds can be kept at bay or rendered harmless. This subject must be reserved for a future lecture under the heading of "Aseptic and Antiseptic Surgery," for it is too big a one to be brought in here save in the most cursory manner. We shall have to go thus far with it, however. We shall have to point out that these micro-organisms are highly infective in their nature; that wherever they gain access to the body they have a remarkable power of multiplying themselves, and of producing deadly changes in the parts in which they have taken up their abode, and that they are so horribly infectious that wherever they can live and multiply they set up new attacks of disease similar to the disease from whence they sprang. They breed true, that is, just as the grain of wheat when sown produces other grains of wheat, so the micro-organism of pyæmia or diphtheria, when sown in the human body, produces fresh attacks of pyæmia or diphtheria when it has managed to "take root" and flourish. See now how the definition "seed-bud," as applied to a germ, adequately expresses what we want to say of it.

Regarded from this point of view, of a poisonous deadly agency, germs are spoken of as septic or poisonous bodies, and the branch of surgery which deals with them is spoken of as aseptic (absence of poison) or antiseptic (opposed to poison) surgery. Those who study disease are compelled, however, to recognise the existence of bodies which, whilst they poison the system and set up inflammations within it, do not multiply themselves in the body, and so produce effects which are proportionate only to the amount circulating in the blood. I wish you to understand this matter clearly. In the one case a seed-bud, a germ, a poison, has gained access to the blood: it has now found a fluid in which it can live and multiply; and, living and multiplying there, it has a means of keeping up, and freshly starting, new disease of its original type; in the second case a poison capable of setting up disease has gained access to the blood, but the effects it produces are strictly proportionate to the amount of it which has got there, and it has no power of re-originating and multiplying itself. Hence it is customary to speak of two kinds of poisons as acting in the body; the septic ones and the infective ones. They are both really septic agencies, but the ones which do not multiply in the blood are due to putrefactive changes in fluids, whilst the others are due to the action of living and birth-giving germs.

### Post-Graduate Clinics for Nurses.

By a Trained Nurse.

XXIV.—A TYPICAL REST-CURE (*continued*).

In rest-cure treatment very little attention is directed towards treating the digestive disturbances as such. These complications are referred to a deranged nervous system, and as the nerves become quiet and soothed, most allied aches and pains disappear or are modified. It is really wonderful to note how "rest patients" are relieved of their many liver symptoms—furred tongues, indigestion, and so on, merely by lying quietly in bed. The regularity of diet and the simplicity of their food, coming after constant self-drugging for dyspepsia, has of course much to do with the improvement shown after a few days' rest.

Mrs. V. had a history of long neglect of dietetics, and to this her medical adviser attributed in a great degree her persistent sleeplessness. She had the usual complaints to make of "soreness everywhere," backache, headache, and mysterious fluctuating pains all over.

She was put to bed in a small ward wherein one other patient was undergoing a similar *régime*. She received the usual instructions to preserve a rigid silence, to avoid moving or turning over in bed without the aid of the nurse, and sitting up was rigidly forbidden. She was not allowed to wash her own hands, to dress or brush her hair, and she was to be "spoon fed"—that is to say, every morsel of food she took was put into her mouth by the nurse who was told off to look after her and the other occupant of the ward. She was put on to the ordinary house diet, with the exception of red meat, which in her excitable, irritable condition the doctor crossed off her diet sheet.

House diet began by a breakfast cup of chocolate made with milk at about half-past six a.m. In those cases where constipation proved unusually obstinate a small cup of black coffee was substituted for the chocolate. And in most instances the effect was admirable. After the early cup of chocolate or coffee the patient was induced to lie quietly and to take a nap if anyway possible. After which a sponge bath was given, the patient lying horizontally and being wrapped in the ordinary way in a blanket. Some patients found the daily bath curiously tiring; in these cases it was given on alternate days. Other patients, too, found that the sponge bath given before breakfast, in spite of the cup of milk chocolate, made them "feel all cold and used up." So that it sometimes was necessary to give the bath at about midday. Then again, for restless, irritable, and sleepless patients it proved better to give the warm sponge all over in the evening for the purpose of soothing the nerves and sending the patient to sleep.

Mrs. V—— being obstinately sleepless was ordered her sponge bath in the evening, and in addition she took five grains of bromide of potassium four times daily.

The house breakfast at eight a.m. consisted of oatmeal porridge, cracked wheat or some other cereal, with eggs boiled or poached, chops, steaks, or fish. Stewed apples or stewed prunes were always served with breakfast, the patient drinking either milk or cold water. In some rare cases hot milk or hot cocoa was allowed. The patients were encouraged to take a good deal of butter with all their meals. Dinner at one p.m. consisted of meats of various kinds, or chicken with vegetables of several varieties, light custard or rice puddings, stewed or fresh fruit, and again milk or water. Supper at about six p.m. was made up of light dishes such as cold chicken or tongue, custards and nourishing but delicate puddings, with plenty of stewed fruit, biscuits, and many different kinds of bread. Curiously enough—and this one particular will well illustrate the difference between American and our dietetics—most of the patients were allowed to eat very fresh bread. Daily dietetics are certainly better understood in England than elsewhere. But the higher branches of dietetics for severe illnesses have been carefully studied in the United States, as is shown by their admirable diet and cookery kitchens, and by the perfection of their peptonised and other invalid foods. In addition to the house bill of fare set forth nearly all the patients were in the habit of taking about two quarts of milk daily, between times.

A few days' trial revealed the fact that Mrs. V—— was unable to cope with ordinary diet, so she was put upon milk only. She began with three quarts in the twenty-four hours, but, her digestion still showing signs of failure and inefficiency, she was put on peptonised milk—forty-eight ounces in the twenty-four hours—to be gradually increased at the rate of a tumbler a day until she was taking the full three quarts.

Dr. Weir-Mitchell always lays stress on the fact that the digestibility of milk depends largely on whether it be taken slowly or quickly. "Rest patients," he is wont to insist, "should be encouraged to spend half an hour in drinking a tumbler of milk. A glass of cold milk quickly taken into the stomach is apt to resolve itself into a hard, indigestible mass of curd. Taken mouthful by mouthful, the state of things is quite different. And nearly all those patients who assert that they are quite unable to digest milk in any form find they can easily cope with it when slowly sipped."

The usual plan is to arrange for the resting patient to take liquid foods through bent tubes. Her constipation proved very troublesome, and she was ordered one teaspoonful of bran to be taken three times daily in a glass of milk. In addition, two drachms of castor oil were administered every night.

An unusual order was made in her case that she was to have massage for fifty minutes daily. The average rubbing for a rest-cure patient in the hospital was twenty minutes. But in private practice some patients receive the full hour. In Mrs. V.'s case massage was not given because she was emaciated or, as is so often the case, to counteract the wasting of the muscles, which is so common an accompaniment of nervous prostration. On the contrary, she was a very muscular woman and her muscle fibres were firm and strong, quite contrasting in this with the soft, flabby, feminine muscles of the majority of her worn-out sisters. Massage in this large dose was given to "weary her out," and to induce enough bodily fatigue as to enable her to sleep.

The bran and castor-oil acted very satisfactorily in relieving the constipation, but "drugging" of all kinds is discountenanced in rest-cure, especially as most such cases have been for months and even years making themselves the receptacles of samples of all and sundry of the stores in the chemists' shops. So after Mrs. V. had been on rest treatment for some ten days, the aperients were stopped in order to test how far the bowel had regained its normal power.

### Death in Our Ranks.

At the Edinburgh Royal Infirmary, on July 19th, 1897, Jessie Blanche Wood entered her rest, within three months of obtaining her three years' certificate, at the completion of which she had hoped to go as a medical missionary to Turkey. All skill and kindness was bestowed upon her. A service was held in the hospital. The matron, medical staff, nursing staff, and others were present, who brought beautiful wreaths, and several accompanied the funeral to the Grange Cemetery on July 22nd.

### Royal British Nurses' Association.

H.R.H. the Princess Christian of Schleswig-Holstein was not present at the annual meeting of the Royal British Nurses' Association, and fortunately so, as the meeting resolved itself into one of the wordy battles of the internecine war that has for the past few years distracted the Association and done it grievous harm in the estimation of the public, and in that of the general body of nurses.

The annual report of the Executive Committee was read by the Hon. Medical Secretary, and agreed to, and the financial statement made by the Hon. Treasurer was received with applause, well merited for the capable way in which the pecuniary affairs of the Association have been wrested from the verge of bankruptcy and placed upon a solvent basis.

The chief bone of contention was the recognition by Mr. Fardon, as Hon. Medical Secretary, and not on behalf of the Executive Committee, of a series of charges which have been formulated against the officials of the Association, and which have been recently published. Mr. Fardon read a lengthy defence of the officials' action (of which we give a summary in another column), but he ought on this occasion to have been the mouthpiece of his committee, and to have had the loyal and strong support of his colleagues.

The whole difficulty, according to the chief speakers against the officials, has arisen owing to the vague wording of one of the bye-laws, which provided that one-third of the members of the council should retire annually, and should not be eligible for re-election for the space of a year. Certain matrons who had worked hard for the Association's charter held themselves exempt from the ruling, and felt hardly used when legal advice accorded with the opinion of the committee that they must retire in their turn. Instead of obeying with dignity and offering themselves for re-election at the expiration of the prescribed period, when doubtless they would have been welcomed cordially, they chose rather to ascribe the decision to personal animus on the part of the medical men who were members of the committee, and have descended to such insulting and unrestrained personalities as to alienate the sympathy even of those who best knew any good and earnest work they may have done.

With regard to the vexatious and frequent lawsuits which have sapped the energies and weakened the resources of the Association, on which so much stress has been laid, two points should be noted. One, brought out by Mr. Fardon, is that the Association has never been the plaintiff in the suits in which it has been involved. The other is that, with the exception of probably five members, the whole are agreed that all differences should be settled by private and friendly discussion; or, failing that, should be referred to arbitration and not taken into the law courts.

The one point conspicuous at the meeting, one which has been noticeable at the meetings of the Association for a long time past, is the terrible waste of time and energy in frivolous disputes, and unless the managing committee pull themselves together and insist upon the meetings of the Association being carried on in a serious and methodical manner dissolution must be the end of it.

### THE ANNUAL MEETING.

The annual general meeting of the Royal British Nurses' Association was held at the Imperial Institute on Thursday, 22nd inst. The meeting commenced at eleven o'clock for the appointment of scrutineers of the ballot papers for the election of members of the General Council, after which it was adjourned until twelve o'clock. On reassembling Sir James Crichton Browne (in the absence of the President, Princess Christian) took the chair.

The minutes of the last annual meeting having been read, Dr. Bedford Fenwick insisted on an addition being made to that portion of them which referred to the Breay incident, bringing out the fact that Miss Breay had handed the Chairman a receipt for a registered letter which she stated referred to the letter containing her resolution.

After a brief but stormy discussion, the alteration was accepted, and the minutes, subject to the addition, were adopted.

### The Medical Secretary's Defence.

The Hon. Medical Secretary (Mr. E. A. Fardon), in moving the adoption of the report of the Executive Committee, craved the indulgence of the meeting to read a statement (as he had been authorised to do by the Executive Committee) concerning the charges which had been made against the officials of the association.

Mrs. Bedford Fenwick wished Mr. Fardon to make it clear to the meeting that the statement he was about to read was drawn up by himself, and was merely his personal opinion and statement.

The Hon. Medical Secretary then read a statement replying to the charges contained in a protest emanating from the Central Council of the Incorporated Medical Practitioners' Association, signed by the president and secretary of that body, and two other documents signed by matrons and nurses. The charges, he said, were many of them couched in vague and intemperate language, which rendered it difficult to deal with them, but, so far as they were definite and distinct, they might be stated as follows:—(1) "That in 1895 the officials, by a quibble, removed certain matrons from the General Council," to which he replied that when it was found legally impossible that these matrons should retain their seats under the present bye-laws, it was decided to reconsider b ye laws. This was now being done, and it would be found the new bye-laws did confer a large number of ex officio seats on the council upon matrons of important institutions. (2) "That effect had not been given by the Executive Committee to promises alleged to have been made by certain members in their private capacity." He pointed out that the meeting at which these promises were made was an informal one, without the cognisance of many of the Executive Committee, and its decisions, therefore, could not be binding on that body. (3) "The action of the Executive Committee with regard to Miss Barlow." It did not appear necessary to reopen the discussion on this subject. (4) "That the Association had for the last three years been mismanaged, its expenditure being allowed to largely exceed its reliable income." As the financial management of the institution was the ground of an action for libel now pending it would be improper to discuss it at present. (5) "That the Association was practically controlled by five medical men; that opposition and criticism were stifled, and free discussion was not allowed." No facts were given for this statement, and Mr. Fardon denied that there were any grounds for the assertion. (6) "That the General Council was packed with nurses from the Middlesex Hospital and the Chelsea Workhouse Infirmary, and that the officials had thus prepared for themselves a majority which was practically compelled to adopt any proposals they might choose to make, and that the paid servants of a public institution were employed by their superior officers for the fulfilment of private ends." In reply Mr. Fardon pointed out that the General Council was composed of more than 300 members—100 medical men, and 200 matrons, sisters, and nurses, besides vice-presidents and a few other ex-officio members. The 100 medical men and the 200 matrons and nurses had to be elected every year, and by the action of the bye laws nearly 70 matrons and nurses retired annually, so that this involved the election of at least 70 fresh matrons and nurses every year. Having recited the circumstances under which the General Council was elected he said the Middlesex Hospital was represented by only sixteen nurses in the service of the hospital, and the Chelsea Infirmary by five out of the full body of 200. He emphatically denied that any use whatever was made of any members of the council to promote the private ends of the officials. (7) "That new bye-laws had been drawn up by the officials without consulting the members, and which they had even prevented the Executive Committee from discussing." This statement was absolutely without foundation. (8) "That the journal of the Association was used by the officials to publish personal attacks against their opponents, and that letters written to the journal in defence were suppressed." No instances were given of this charge, and a reference to the journal of the Association would prove how groundless it was.

After reading this statement the Hon. Medical Secretary said there seemed to him to be something of more importance to nurses than the question whether one person or another person should occupy an official position on the Association, and that was the effect of these unnecessary contentions, not only on the Association but upon the public mind, and so upon nursing generally. (Applause.) If, unfortunately, the Association should prove to be the means of fostering strife and angry feelings amongst the members, if it should be the means of inducing members to write bitter articles in the public papers against one another, or to resort to litigation, he did not hesitate to say that the effect upon the spirit in which nurses should carry out the great work of their lives would be so disastrous and so harmful that it would have been better for nurses and nursing, for suffering humanity itself, if the Royal British Nurses' Association had never been founded. (Applause.) He was, however, sure that the very great majority of the nurses who formed the Association were pained and astonished spectators of the unworthy attempts to sow discord in their midst. (Applause.) He begged them not to allow themselves to be drawn into these quarrels—(applause)—but rather to help to restore order and peace—(applause)—so that the Association might be free to carry out and develop the true purposes for which it was founded. (Renewed applause.) Owing to these agitations the progress of the Association had been impeded, its resources squandered, and it suffered in public estimation—("Hear, hear" from Mrs. Bedford Fenwick)—but he appealed with every confidence to the common sense and high-mindedness of the nurses who formed the large majority of the members to support those who were doing their very best to bring about a happier state of things. (Loud applause.) He then read the report of the Executive Committee and formally moved its adoption.

Mrs. Coster seconded.

Mrs. Bedford Fenwick read a lengthy indictment of the present management of the Association, in which she said that, there being no hope that grievances would be redressed from within the Association, a public inquiry had been demanded. If the honorary officers shirked that inquiry the public could only draw the conclusion that they were afraid to face it. (Laughter and interruption.)

The Chairman called for order.

Mrs. Bedford Fenwick (speaking amidst renewed interruption) said she had a great deal more to say, and intended to say it. (Renewed interruption.) Continuing she said that in order to explain the present position of affairs she must go back some twenty years, at which time the internal management of hospitals and the nursing of the sick especially was in a chaotic condition. Educated women, however, gradually took up nursing as a profession, and reform took place rapidly, but it was not until 1887 that the women who had survived the ordeal of this pioneer work, and who then found themselves in the leading positions of their profession, met together at her house—(laughter)—to discuss the vital question of how the vocation of nursing performed under a terrible physical strain could be organised into a clearly defined profession, which would give to its members the protection of a legal status, and in consequence make it possible to organise and improve their conditions and labours. On her suggestion it was decided to form the British Nurses' Association. It was the nurses who took the initiative, and medical men were asked to assist them to attain their end. The women who had founded the Association worked together early and late, until in 1893 the Association obtained its Royal Charter, and from that date the present troubles began. They all knew the proverbial characteristics of the cuckoo. He was not a desirable bird, and he had his human imitators. When the hard work was over, and success seemed assured, persons who had previously opposed them, or who had stood aside during the struggle, joined them. Dr. Bezly Thorne who was then honorary medical secretary—(applause and hisses)—was the author of a very undesirable effort at dictation on the part of the medical members which crept into the work of the committees. He also discovered an ambiguity or flaw in the bye-laws, which implied that the matrons who took an active part in founding the Association, and who were promised permanent seats on the General Council, must retire from it. A requisition for a special meeting to obviate this by altering the bye-laws was signed by members, but was ignored by the Executive Committee. These matrons were accordingly compelled to retire, and, being naturally indignant, determined to make a public protest at the annual meeting of 1895. However, several of them were asked by some of the medical members to meet them, and, in consequence of the promises then made them that their grievances should be redressed, no such protest was made. When fulfilment of the promises was asked for, they were calmly repudiated, and as a result of this breach of faith the Association had gone steadily downhill in public estimation ever since. Then came in 1896 what was known as "the great betrayal" of the Association, when Miss Wedgwood and Mr. Fardon (the honorary medical secretary of the Association) had voted at a meeting of the British Medical Association against the State registration of nurses. Finally, in October, 1896, came the proposal, made by the honorary officials, to add male and female lunatic attendants to the register of the Association, and to admit them to the full privileges of membership. (Applause.) Of the personal intimidation, from which she herself and many other matrons and nurses had suffered, she would say nothing, because they were fully determined that this system should not prevail. They would take no statement as final concerning their accusations against these persons—(laughter)—but they demanded a public inquiry into the affairs of the Association, and they were prepared—(interruption, in which the remainder of the speech was lost).

### A Challenge.

Miss Helen Foggo Thomson, as an independent member, who had had much to do with the internal management of the Association during the time it was controlled by Dr. and Mrs. Bedford Fenwick, desired for the guidance of the members of the Association, and for the information of the Press, to ask Mrs. Bedford Fenwick a question. She hoped that the question would be answered frankly and straightforwardly.

Mrs. Bedford Fenwick (excitedly): These are the ordinary courtesies of debate, I suppose.

Miss Foggo Thomson, without noticing the interruption, continued that it had seemed to her that there must be some simple cause for those interminable personalities which had taken place at the meetings during the period since Dr. Bedford Fenwick resigned the post of treasurer, and that was why she put the question. She desired to ask Mrs. Bedford Fenwick whether it was not a fact that on more than one occasion, and to more than one person, she had stated in effect that she had made the Association, and that if she was not permitted to control it she intended to destroy it, for no one else should conduct its affairs without her? (Cries of "No, no," and hear, hear.)

The Chairman said that Mrs. Bedford Fenwick was not obliged to answer that question, of course.

Mrs. Bedford Fenwick very excitedly said she presumed this was what they called fair debate. She was not obliged to answer Miss Thomson, but she would tell her that she was stating an untruth. (Cries of "Withdraw," groans, and hisses.)

The Chairman called upon Mrs. Bedford Fenwick to withdraw that remark.

Mrs. Bedford Fenwick: I call for written evidence for these accusations against me; I call for witnesses or documentary evidence of what she states in that question. That I had the honour, amongst other matrons, of helping to form this Association, I am proud—not of the Association of to-day, but of the principles on which it was founded—but I shall never submit to what I know to be unjust, and if, by not submitting to what is wrong and unjust, the Association as it is now controlled is wrecked, that is not my fault. ("Oh, oh!")

The Chairman said that while allowing the fullest latitude to the debate, he could not approve of Mrs. Bedford Fenwick's accusing any member of the Association of a deliberate untruth. He was sure she had spoken on the spur of the moment. (Interruption.)

Mrs. Bedford Fenwick: No, not at all.

The Chairman: At any rate she will withdraw it.

Dr. Bedford Fenwick interposed, and congratulated the Chairman upon his asking that the words "an untruth" should be withdrawn, when at the last meeting of the Council he had permitted Dr. Bezly Thorne to say that a statement was infamous and blackguardly. He called for evidence of the accusations made by Miss Thomson.

Miss Foggo Thomson: I ask Mrs. Bedford Fenwick to deny my statement. Do you, Mrs. Bedford Fenwick, deny that since you have returned from Greece you have stated to members that if you spent your last cent you intended to employ the strongest counsel you could get and smash the Association?

The Chairman: This incident must end.

Dr. Bedford Fenwick said that this question, without the slightest foundation or evidence, had been brought forward for reasons which were obvious. He had only to say that the public inquiry they demanded would be granted, and Miss Foggo Thomson would then be enabled to produce herself and her witnesses.

The Chairman said that the inquiry referred to had been asked for and refused. If the Home Secretary and the House of Commons were to institute an inquiry into the affairs of every association where the minority did not agree to bow to the majority they would have their hands full.

Miss Lee, Eye and Ear Infirmary, Dublin, read a statement, which she said she made on behalf of other matrons of leading Irish hospitals, reproducing most of the charges already referred to in the previous portions of the discussion.

Mr. Hugh Woods, President of the Incorporated Medical Practitioners' Association, replied to the answers made by Mr. Fardon to the eight charges Mr. Fardon said had been brought against the Association. He thought the discussion which had already taken place was quite sufficient to justify his association in the action they had taken—action which was solely and entirely taken in the interests of the Royal British Nurses' Association. For many years it had been notorious that the work of the Association had not been carried on in a manner which could conduce to its welfare. They had waited for a long time, hoping that the Association itself would bring about a better state of things, but instead of that they saw things going from bad to worse, rows at meetings, strong methods to check debate, law cases, and so on, increasing instead of diminishing, until it seemed likely that the Association would become a sort of public bear garden for the amusement of those who delighted in such things.

### The Promise to the Matrons Explained.

Mr. F. Pickering Pick explained that the meeting of the matrons which had been referred to was held in his house, and was summoned by him. No promise was made at the meeting beyond the promise of some of the members that they would do their best to get a revision of the bye-laws. It was distinctly stated that that promise was made by them as private individuals, that the meeting was a private and informal one, and that they could not bind in any way any other members of the Executive Committee.

Dr. Bedford Fenwick said that he was glad to hear that contradiction from Mr. Pick. However, twelve matrons of leading hospitals, who had been determined before going to that meeting to express their views at the annual meeting, left that meeting believing that they had gained the redress of their grievances. Referring to the statement read by Mr. Fardon, he said the whole origin of the trouble was connected with Dr. Bezly Thorne, who was the author of the suggestion that the matrons who had founded the Association should be removed from the General Council. The statement he characterised as the most forcibly feeble thing he had ever seen. It did not deal with one single serious charge, and was not worth the paper it was written on. After reiterating the fact that a public inquiry would be held, he said that they were now only at the beginning of the movement which would take place in every part of the country until the inquiry was demanded.

### The Closure Applied.

The Chairman said that, while unwilling to put any restriction on the discussion, he thought the matter had been sufficiently discussed. He would take the sense of the meeting on the point.

Miss Breay asked Mr. Pick whether the gentlemen at the meeting at his house had done their best to obtain the fulfilment of the promises then made?

Mr. T. Pickering Pick said he could only speak for himself. He could say that he had done his best. (Applause.)

The Hon. Medical Secretary, in replying to various statements made during the discussion, said it had been suggested that the officials were shirking a public inquiry. The officials, he said, were the people who, of all others, would desire the fullest public light being thrown on their doings—(applause)—but the question as to whether a public inquiry should be held did not rest with them, but with the public, and the people who represented the public. He also explained that when, as Mrs. Fenwick had stated, he attended the meeting as to the State registration of nurses, he did so, not as the hon. medical secretary of the Association, but as a delegate from the board of his hospital in order to represent the views of that board on the subject. Finally he pointed out that in all litigation which had taken place the Royal British Nurses' Association had been the defendants.

The adoption of the report was put to the meeting, and carried *nem. con.*

### The Treasurer's Report.

The Hon. Treasurer (Mr. J. Langton) presented his financial report for the year ended March 31st, 1897, and in moving its adoption appealed to all to bury the hatchet, and give comfort and ease, at any rate to their treasurer. (Applause.)

Dr. Calvert seconded, and bore testimony to the zeal and energy of Mr. Langton, to whom the Association ought to be very grateful.

Dr. Bedford Fenwick commented on the report, which was unanimously adopted.

### Election of the General Council.

Dr. Calvert presented the report of the scrutineers, which stated that 632 of the ballot papers had been returned unaltered, 80 had been altered, 12 were unsigned, and 32 were rejected because of arrears of subscriptions.

The Chairman declared from the chair that he General Council as proposed by the Executive Committee had been elected by an enormous majority.

### Vote of Thanks to the President.

Dr. Bezly Thorne moved the following resolution: "That this general meeting tenders to H.R.H. the Princess Christian of Schleswig-Holstein the expression of their respectful duty and their grateful appreciation of the solicitude which Her Royal Highness has always evinced for the welfare of both medical and nursing members of the corporation." (Applause.)

Miss Wedgwood seconded the resolution, which was carried unanimously.

### Vote of Thanks to the Officials.—Another Storm.

Dr. Buzzard said it was his pleasing task to propose a cordial vote of thanks to the honorary officers. (Applause.) It appeared to him that the so-called charges which had been brought against them were really of a very trivial description. [Mrs. Bedford Fenwick: "No, no!"] Had they even been subtantiated to the hilt [Mrs. Bedford Fenwick: "Give us the chance!"] they could not possibly have justified any serious condemnation of the honorary officers. As it was, it appeared to him—and he had listened attentively to all that had taken place—that these charges had utterly broken down. (Applause.) [Mrs. Bedford Fenwick: "No, no!"]. And he could not imagine any dispassionate listener feeling otherwise than satisfied that these charges, so offensively circulated, whatever might have been the motives by which those who had brought them were actuated, did not represent any real solicitude for the interests of the Royal British Nurses' Association. (Loud applause.) There was something to his mind positively revolting to one's sense of justice—[Mrs. Bedford Fenwick: "Hear, hear; sense of justice!"]—in the idea that members of the Association, who had kept up the laborious and thankless task of carrying on the work of the Association, should have been singled out for attacks which were as vexatious as he though they had been proved to be baseless. (Applause.)

Dr. Bedford Fenwick: I rise to order.

The CHAIRMAN: I am the chairman, and will regulate the order of the meeting if Dr. Bedford Fenwick will allow me. (Applause.)

Dr. BEDFORD FENWICK: I rise to order.

The CHAIRMAN: I call upon Dr. Buzzard to proceed. I think this meeting will agree with me that Dr. Bedford Fenwick has had a sufficient hearing, and we must proceed. Dr. Buzzard is in possession of the chair. (Applause.)

Dr. BEDFORD FENWICK still persisted in addressing the chair, in spite of interruptions from the audience, saying that no resolution could be proposed at that meeting without notice having been given.

The CHAIRMAN: This is a vote of thanks.

Miss BREAY rose amid cries of "Order!"

The CHAIRMAN: I cannot hear you at this moment. Dr. Buzzard is in possession of the meeting. (Cheers, hisses, and continued interruption.)

Dr. BUZZARD, continuing, said that, from the experience he had had on the committees, he could unhesitatingly testify that the example of untiring energy set them by the president was worthily followed by the honorary officers, who had given their time, energy, knowledge, and in many cases their money, to promote the welfare of the Association. (Applause.) In view of what had taken place that day, he was convinced that there would be an overwhelming expression of opinion that the honorary officers were entitled to their strenuous support, that the charges made against them had fallen to the ground, and that they deserved the warmest sympathy at the hands of every right-minded member of the Association. (Loud applause.) He then proceeded to move, amidst continual interruption from Dr. and Mrs. Bedford Fenwick the following resolution: "That this meeting, having heard the reply which has been made to charges against officials of the Royal British Nurses' Association, desires to put on record its continued confidence in the honorary officers of the Association and the Executive Committee, to express its sympathy with them in the annoyance to which they have been subjected by the publication of vexatious and unfounded accusations, and to accord them its warmest thanks for their most valuable services." (Loud applause.)

The CHAIRMAN said that he was of opinion it would be better to restrict the motion to a simple vote of thanks to the honorary officers. In voting for it, however, the honorary officers would well understand that the meeting homologated what had been said by Dr. Buzzard. (Applause.)

Mr. R. BRUDENELL CARTER, in seconding, said that when asked to do so he had entertained the hope that the vote would have been unanimous, because, although he was aware that there were differences of opinion as to the methods of the officers, he did not think there could be any differences as to their having been guided by a desire to promote the efficiency of the Association, and that they were therefore worthy of the thanks of both those who agreed and those who disagreed with them. He thought that an immense flood of light had been thrown that day upon the reasons why some persons amongst the members were unwilling to give this vote. It had transpired that during the past year these officials had succeeded in various ways in so reinforcing the funds of the Association that it had not been possible to wreck it by frivolous and vexatious litigation. (Applause.) He thought that fact alone—the discovery of these outside sources of income, and the defeat that had consequently fallen upon the plans of those who expected to reduce the Association to a state of penury—was a statement which thoroughly merited the thanks of the meeting. (Loud applause.)

Mrs. SOMERVILLE asked, if this was really a British Nurses' Association, why there were no nurses to propose these votes of thanks?

The CHAIRMAN suggested that it was because nurses, with a modesty suitable to their sex, were a little reluctant to speak at the kind of meetings the Association usually had. He then put the vote to the meeting, and declared that 104 voted for it, and 9 against.

The HON. TREASURER returned thanks, and proposed that Mr. Hardy be appointed auditor.

The HON. MEDICAL SECRETARY seconded, and it was carried unanimously.

VOTE OF THANKS TO THE CHAIRMAN.

Mrs. SOMERVILLE: May I ask a question?

The CHAIRMAN: Dr. Alderson is in possession of the chair.

Dr. ALDERSON moved a vote of thanks to the chairman, which Miss THOROLD seconded, and which was carried *nem. con.*

The CHAIRMAN, in returning thanks, said that all the honorary officers were in complete accord, and any attempts to cause divisions in their ranks were in vain. He had been accused of serving private ends. Had he been seeking them, he would have aimed at a vote of censure that afternoon, which was just what would have relieved him of his difficulties. He reminded his opponents of the fact that threatened associations, like threatened men, lived long. (Applause.)

The meeting then broke up, Mrs. SOMERVILLE plaintively shouting, "May I not ask my question, please?"

### A Cottage in the Country.

"WELL, lass, and where have you been gadding to all this day?"

"Oh, John, I hardly like to tell you; but I am so glad. I've seen my girl."

"Now don't bring that up again. You know she has disgraced herself and has dropped out of our life. Don't trouble about her any more."

"But, John, I've seen her. The postman brought a letter, after you were gone, from one of the sisters at the hospital saying how ill she was, and that she had told her true name and had asked to see her mother. What could I do, John? I just went off, and when I got there I saw the sister, who told me all about it. She knew all the disgrace; she knew all that she had done; but she had taken care of her, and she had been kind to her, and why should not we be kind to her again now that we have found her? There was not a thing she had not told to that good sister, yet she had not shrunk from her, and why should we? No, John, she is ill and weak, and she is very sorry, John, and I am going to bring her home next week."

And so peace fell on a household where all had been darkness since the pet lamb had strayed away.

### Antique Safety Pins.

A MOST instructive portion of the Medical, Surgical, and Hygienic Exhibition is the collection of old surgical instruments and nursing contrivances, some dating from 3,000 years B.C., shown by Messrs. Oppenheimer. Among them are two specimens, aged 2,000 years, of the genus safety pin which was supposed to have been invented some 50 years since by Newnham, of Birmingham. This ancient pin is made on exactly the same principle as that which the nurse of to-day carries in her chatelaine, but the outside bar, instead of running parallel with the pin, rises in a curve as of a bent bow, the pin forming, as it were, the string.

### Novelties for Nurses.

### LOCKERS AND CHARTHOLDERS.

THE National Hospital for the Paralysed and Epileptic had new lockers last year in the "Princess Christian" ward. They are made entirely of polished wood, and have a drawer at the top, two small cupboards opening in opposite directions in the middle, and a large cupboard at the bottom. The back is well ventilated and provided with a towel rail. The nurses like them very much, as they are easy to move and look well. The chartholders are also of last year. They too are of polished wood, with a wooden edge on hinges, somewhat resembling a slate. The medicine chart is put in front, and notes and directions are put on the chart at the back.

### The Etiquette of Nursing.

By G. A. Hawkins-Ambler, F.R.C.S.E., Hon. Surgeon Samaritan Free Hospital for Women, Liverpool; Author of "The Gentle Art of Nursing the Sick," &c.

I wish some competent authority would write a book on etiquette for nurses as excellent as the code of ethics drawn up for doctors. If this were accomplished, it would afford much satisfaction to the medical profession, and, if acted upon, would immensely increase the appreciation of the nursing profession. Every practitioner who has to deal with nurses, in private or hospital practice, knows how much he owes to them, and never ceases to bless the tribe—except for the purpose of cursing those members of the race who are deficient in etiquette, or who have not the advantage of being gentlewomen and able to do without it, or Christians, and so summing it up in the Golden Rule.

What are the relations of the nurse to doctor and patient? I never write or speak about nurses without making trouble for myself, so that I shall only attempt here to indicate one or two sources of dissatisfaction, and possibly a suggestion for remedying them.

I have absolutely no experience of male nurses, so that I may be wrong in attributing the faults of nurses essentially to their sex. But woman is the prey of her enthusiasms: she is prepared to sacrifice herself and all she holds dearest and highest to the dictates of love and the call of friendship, but she cannot see both sides of the question in such a case. She has the faults of her qualities, the limitations of her sex, and its ignorance of life and affairs. Common business honesty, the etiquette of a profession, she is too apt to scorn or ignore. She has a friend, she must move heaven and earth to help him; she has an idea, that idea has to be forced on the world; she is at some pains to grasp the lesson she is taught, and she holds the teacher the one authority, his method the only one. Now such things are all very well for woman when she is not placed in the position of servant to many, and with the interests of many persons and diverse cases to deal with.

Take a nurse who has been taught in the wards of a physician who believes that the ice pack is the best treatment for pneumonia. Picture this enthusiast passing into the world. She will soon be nursing for men who never heard of the treatment, others who hold it to be fatal, and others, again, who believe firmly that the linseed poultice is the only permissible mode of treating such cases. I am prepared to anticipate a warm time for the doctors that nurse will work for. She will feel morally bound (and this is the worst of the whole business) to protest to her patients during the whole of the twenty-three hours and fifty minutes of the doctor's daily absence from the bedside, that they are being destroyed by an ignorant and incapable man. If haply she find that a case recovers in spite of a poultice, it will be ascribed to the Providence of God; while if she happen to nurse several cases that make so unexpected a recovery, she will run the risk of becoming a medical sceptic, unless she get her experience rapidly and wisely, and so find out what a sensible woman does in time—that there is a good deal more in medical science than she has dreamt of, that the snuffy country practitioner may possibly know something of a profession he, too, learnt in the hospitals and has been practising day and night for twenty or thirty years, and that she is an ignorant and futile little wretch for overbalancing her mind with only one set of facts, and so thinking through life lop-sidedly. We all have our fads. The trouble is that a nurse is usually trained on one person's fad, and takes the fad for science, seizing with a woman's thoughtless enthusiasm and instinctiveness on the unnecessary and worthless in place of the abiding and the true. A nurse, however, rarely thinks how wrong she may be, and if so, how wicked in upsetting confidence that has been well earned by faithful and intelligent service, because the doctor's ways are not the nurse's ways. Why should they be her ways? Who, after all, is master? A doctor may be rusty in the new ideas of the schools, he may be careless, tired, overworked, not so bright as he might be, but he has been trained for a longer period than the nurse, his education, at the worst, is infinitely better. While the nurse is watching one case she has not been taught to understand, the student is studying a score that probably show all varieties of treatment, exhibited with an almost equal success throughout. The nurse is apt to think with the patient, that the particular case she is interested in is the only one calling for the doctor's attention, and to forget that he may be attending a dozen like it which he mentally compares and studies together. The most experienced nurse is only experienced in nursing, not in disease of any range. She certainly has a closer intimacy with her particular case, sees every change; over-estimates every symptom probably. Over-estimates, I say; for though her closer observation may be of great value, there is no doubt that for her symptoms and signs grow distorted. I know doctors who would be terrified if placed in very close attendance on any case. They would not trust themselves, they would lose their balance, misplace symptoms in importance, and disarrange facts in the inverse order of their proportion. The nurse loses this anxiety in her duty to the doctor. That duty is to obey him and recognise his sole responsibility for treatment, and her own only responsibility as an executive officer. Rightly or wrongly, we cannot have every subaltern of genius discussing his superior's orders. Only one battle has been won in a century by the disobedience of orders. But let not the nurse think herself a Nelson!

Inexperience, and not insight, is the origin of many of our troubles with nurses. We don't place any value on mares' nests as we grow older and see more of the world; but I acknowledge they play an important part in the discoveries of youth. An intelligent doctor values the suggestion of the youngest tyro if made with proper respect, and, if a gentleman, treats it with consideration. But it is not the place of the nurse, surely, to discuss treatment with patients: rather give me indifferent treatment with confidence in my doctor than the best without that regard that so often saves. But if your nurse says, "Oh—h—h! am I to do so-and-so? Dr. —— always did this, that, or the other," it is calculated to make the doctor consign Dr. —— and his nurse to other quarters. Still, it is done, and the nurse as often resents any improvement the patient has the impudence to make under any other form of treatment than that she prefers.

Another part of some nurses' creeds appears to be the duty of advertising her favourite doctor, or her hospital. Now we do not employ a nurse to advertise another man, or to push her own hospital. We want our patients nursed, and, while we do not want belauding to them, we do not wish to have the confidence we have usually fairly earned undermined by the nurse we have called in to assist us. We send for a nurse in order that our own treatment may be fairly and effectively carried out. This, I urge, it is the nurse's duty to do without prejudice, honestly, completely, without a word of distrust, or a shadow of suggestion in her voice or manner that there is any other treatment possible or any other doctor in the wide world who would or could treat the patient better. Show me the nurse that recognises that as her duty, and I will sell my coat to start a subscription for a monument to her. And why should we not have nurses like that? Is it an unfair thing to ask of a nurse if we try to do our duty by a patient? If the State give us a right to practise, and if we have satisfied Authority that we are qualified to think and practise for ourselves, what business is it of the nurse's whether we meet her views or not? I will not say whose servant the nurse is; it is a question outside this discussion, and as, through a pretty long experience I have come to the conclusion that the nurse is everybody's master, discussion is needless. But for myself I am satisfied that whoever he is the nurse serves, my name and reputation as a man and a surgeon depend on my ideas being carried out as I would have them carried out; unless this is done I can only retire from the case, and leave nurse, patient, and Health Committee to fight it out amongst themselves. That is the position we all need to take—discharge our duty to ourselves and our profession, as nurse or doctor. The nurse is not employed as consultant, as critic, as arbiter; she is strictly an executive officer, and she has no right to carry out on patients either the ideas of herself or the most heaven-inspired hospital surgeon she has had the advantage of being trained under, in preference to those of the responsible medical man on the spot, who enjoys the confidence of the patient he is attending. A nurse who does not recognise this is unfit for her profession, and the sooner she is sent about her business the better for medicine.

But apart from the by no means uncommon interference of nurses with the work of the medical attendant, we have sometimes underhand or tactless ways of damaging the doctor. Recently a doctor employed a nurse from his own hospital to nurse an operation out of doors. Almost the first thing the nurse said to the patient was, "You were silly for having this done at home when you could have had it done by the doctor at the hospital for nothing." The nurse knew that hospitals have a scandalous indifference to the pecuniary position of those who receive attendance at their hands in too many cases, and either from spite or sheer witlessness set the patient about badgering the doctor to lower his very reasonable fee for the operation. Another nurse said, "You ought to have had this done in our hospital" (the institution, that is, to which the doctor sent for this nurse). "Well," the patient objected, "we cannot have our own doctors there." "No, but then our doctors are all very nice." It is probable that her doctors were nice. Had they known the fact that the patient was able and willing to pay a proper fee to her doctor outside for an operation (as well as to pay the institution for the services of the nurses), they would have been too nice to rob him of a patient or to inflict on their charity a case so entirely outside the scope of charity. But such remarks made to mean people, who abound, unfortunately, would be very great inducements to practise fraud in order to save a considerable fee. I am sure that if nurses thought of the littleness of all this, the despicable meanness of it, its unfairness, they would hesitate to let silly tongues wag to so disgraceful a purpose. No nurse who enters a hospital fails to see how they are imposed on by the unworthy, and she ought to be the last to assist, directly or indirectly, in the robbery of the charitable, the needy, and the medical profession. I am sorry that I have not space to enlarge on the subject as I would wish. I can only hope that someone with more time and ability than myself will take it up and write (on the only subject connected with nursing that has been overlooked) a handbook of nursing ethics.

That some such manual is needed all admit who know anything about nursing. The pressure on the medical profession has become so acute, from indiscriminating hospital charity on the one hand, and unfair competition of quacks and cheap doctors on the other, that doctors are uniting in their own defence for the first time in the history of medicine I believe. I am personally satisfied that not only is efficient nursing a necessity of our times, and perhaps the most magnificent development of this great reign from a doctor's point of view; but it must take up its responsibilities as a profession. Nursing is an art, a great art, its votaries are capable of doing immense good and of taking a position in life that is dignified, remunerative, and honourable. We cannot do without nurses; but we shall have to have some control over the unfit.

It is not likely that a doctor will sacrifice the best years of his life to get a qualification, will wait years for practice, in order to have it dispersed by any fool-nurse he chances to call in to his assistance. If nurses will not organise themselves and take steps to control their unworthy members within an honourable calling, or to hound them out of it if incorrigible, the medical profession must. It is a growing evil and a scandal that a doctor cannot have his cases nursed without anxiety lest he is being supplanted by a rival who stands higher in the nurse's regard, or being belittled by one whom he has given the ear of a patient whom tedious illness or querulous disposition make fretful and doubtful, or scandalised by some chattering busybody whose ignorance of her profession gives her the right to criticise a member of a higher calling still. Nursing has achieved an immense impetus from the character and genius of those who in this country have fostered it. As a profession, however, nursing has grown up; it can dispense with the petting, the sweets, the irresponsibility of childhood, and must undertake the cares and the dignity of maturity. And nursing cannot continue to advance—indeed, it will surely retrograde—unless it makes a determined effort to follow ideals that make for righteousness, to recognise the rights of others and its own responsibilities.

### For Better or for Worse?

By a Matron.

So much is done nowadays to drag nurses into the fierce light of public opinion, that they are apt to become either unduly glorified or unmercifully vilified, according to the prejudices of their critics.

A war correspondent wrote the other day, "What war feels like—No emotions and no moral. The strange thing about war is that it is so wonderfully like peace." The strange thing about a nurse is that she is so wonderfully like any other woman. We ourselves expected that a change would come over us when we donned the cap and apron, and on looking back along the vista of past years it surprises us to find we can still claim the old personality, and that our tastes and habits have not materially altered. Sometimes we are even disappointed to find ourselves so much the same; we had expected such a complete *bouleversement* of our natural propensities. We wondered what we should feel like when we found ourselves in a ward full of sick people, what we should do if somebody died, and, above all, what would be the moral and mental effect of our first operation. And yet, when the events happened, they all came as a matter of course, and we felt no radical shock. The patients became a part of our existence almost as soon as we were aware of their presence; while they lived they demanded our attention; if they died we felt it was past human skill to save them, and that they were better off; while our first theatre experience is probably a confused memory of washing up and a crowd of men. Our friends marvel that we can stand a life so full of sadness and horror, but we know that a hospital ward is not sad, and that there are no horrors such as they imagine.

And so time slips by, and perhaps a day comes that we pause and think, "Am I a nurse—do I feel like a nurse—

have I found my vocation?" And then we may realise that a subtle change has taken place. We have imperceptibly glided into a realm of deeper interest, we have peered into the mysteries of disease and been taught to notice every detail of our surroundings, we have watched the struggles between life and death, and we have been brought into close contact with men and women of all sorts and conditions. We discover that after all we are not quite the same as we used to be. This wider knowledge of our fellow creatures, this clearer insight to their sufferings, the swifter perception of their needs, the more intimate knowledge of good and evil—how have they affected us? Probably we have either become more self-sacrificing, thinking ever of our duty before our comfort, or we have become more selfish, made apathetic by the monotony of our work, and our enthusiasm and interest crushed out by the mere desire for independence. Once it was remarked, "Nurses never look old because they are never thinking of themselves." Here is a test. If this is true of us we have found our vocation, and our character has broadened and deepened without our recognising the process or labouring for the result.

### Everybody's Opinion.

[Correspondence on all subjects is invited, but we cannot in any way be responsible for the opinions expressed by our correspondents. No communication can be entertained if the name and address of the correspondent is not given, or unless one side of the paper only is written on.]

### BADGES AND NURSES AGAIN.

"E. R. W." writes: I do not know whether there are any other nurses who agree with me or not, but I for one would scorn to wear a badge which would be supposed to label and protect me as a trained nurse or an honest woman. I would far sooner people found it out through my work, appearance, and conduct. If only nurses walked in the streets like women with a purpose in life and dressed well and neatly they would not be exposed to the danger of being mistaken for disreputable characters or sham nurses. I can only tell my fellow nurses what I tell other women, "It depends on yourself what men take you for."

### DISTRICT MIDWIFERY.

Amy Evans writes *re* "Obstetrics" query (322 in the number for July 17th) as follows: If obstetric enquirer about district midwifery has been delivering poor women without a doctor's supervision it is gratifying to hear that the poor have taken so little advantage of her services as to make it not worth the while of her committee to retain her services. Nearly everyone knows the evil resulting from the use of untrained women by the poor at their confinements. But having both seen and heard of more grievous results from the use of trained midwives now for many years, I think I should prefer an entirely untrained woman to one who has had six months training in midwifery. If a man went in for the same six months' course of training in midwifery, he would not be allowed to set up as a midwifery practitioner, nor to give a certificate that an infant was stillborn as many of our midwives constantly do. Therefore I ask why should a woman be allowed to do it? Some day the country may find out how much of our infant mortality is due to midwives being able to give a certificate for a still-born child, and also how much crime is concealed in this way. Poor women often suffer for years through the want of skilled medical attendance at their confinements. It is, therefore, to be hoped for the sake of the poor mothers of our land (who often do not realise the perils of childbirth) that the time is not far distant when none but qualified medical men and women shall be allowed to attend a woman in her confinement. No one but a doctor can be sure at any stage of labour that serious complications may not arise; and, should abnormalities occur, no one but a doctor is fitted to cope with them, and the want of this skill constantly means death to the poor woman. The amount of suffering caused both at the time and in later life through this want of knowledge of both trained and untrained midwives is, I fear, too little known. It is, therefore, to be hoped that those kind people who are so anxious for the well-being of our poor mothers will procure the services of a doctor and of a trained nurse to work under him instead of providing an inferior midwifery practitioner as a midwife.

### MEN AS NURSES.

"One Who has Worked with Men Nurses" writes: The writer of the article "Men as Nurses" asks, Where are they to be properly trained? If, as I firmly believe, it would be desirable to have men nurses, could not they be trained in workhouse infirmary wards or in the Local Government Board hospitals, thus making use of the rate-supported institutions?

"Truth" writes: I have read the above article with much pleasure, for I fully agree with the author in all that he says on the subject, and if you have not forgotten at the time of Lady Priestley's bitter attack on us, it was the one point I agreed with her about, "To the pure all things are pure," and when one hears of coarse comments on the subject one is inclined to draw oneself up and say *Honi soit qui mal y pense.* Still it can only be nice and pleasanter for both nurse and patient that trained male attendants should be available. I was about thirty when I took up nursing, before that I had travelled about a good deal, and been at different times both guest and hostess of foreign princes and English noblemen, so that you will agree I have seen every aspect of life, high and low, good and bad, and that I may term myself a woman of the world. My father is the most chivalrous of men, and I have been brought up to take the highest view of woman's purity, but at the same time I have learnt to see the frailty of human nature, and whether people wish to acknowledge the fact or not, it is not nice for young nurses to be sent to chambers to nurse young men, and there certainly ought to be male attendants who are competent to deal with serious cases. Naturally when it comes to saving life, a mother, wife, or sister are obliged to send for a nurse, but do not many unpleasantnesses and complications arise? Surely it is almost a foregone conclusion. I myself twice have been placed in such a position that I have had to nurse gentlemen; in both cases it was a matter of life and death, and I stuck to my post, but I suffered horribly both times. In the first case the patient was so nice and refined that the suffering on his side and mine was equal; in the second the patient was coarse and unchivalrous about his idea of women (and unfortunately he was a doctor), and I was obliged to tell him that it was only because his life depended on me that I stuck to my post. Of course afterwards his manner changed into reverence, but was it a pleasant experience to go through? I have made a point of showing you how much experience of high and low life I have seen, both socially and as a nurse (for I have done maternity work and district nursing in the slums) and if a woman of my stamp has found it unpleasant and difficult to steer, how much more so unsophisticated nurses. Therefore to avoid scandal I say most certainly should the hospitals open their wards for male attendants. There is no reason why there should not be a female and a male probationer in each male ward, and as he advances in knowledge and skill the sister and staff nurse can trust him with more responsibility with the heavier and more serious cases of the ward, and I should say that two years' training ought to be sufficient for a man.

### Presentations.

Crichton Royal Institution, Dumfries. — At the Southern Counties Asylum, Dumfries, Miss Kaye was presented by the staff of the female section with a silver basket, "as a token of affection and esteem," on her resigning her post as matron.

### "THE HOSPITAL" CONVALESCENT FUND.

We thank Nurse Emma Butler for her kindly gift of 2s. 6d. towards this fund.

### Appointments.

MATRONS.

THE CITY HOSPITAL, SHEFFIELD.—Miss Florence Ida Turnell has been appointed Matron of this hospital. She was trained at the Royal Hospital of the same city, and in 1893 became one of the head nurses at the hospital, Weston-super-Mare. Since she left this hospital she has filled the following important posts temporarily: Night sister, the Royal Hospital, Sheffield; head nurse, Durham County Hospital; matron at the Children's Hospital, and also at the Jessop Hospital for Women; and lately matron for the City Hospital for Infectious Diseases, Newcastle-on-Tyne.

SOUTHPORT INFIRMARY.—Miss Alice Clark was appointed on July 14th Matron of the Southport Infirmary. She was trained at the Royal Albert Edward Infirmary, Wigan, where, after three years' successful work, she was appointed sister to two important male wards and the eye and ear department. Two years ago she received the post of assistant matron in the same infirmary.

COTTAGE HOSPITAL, BRIDLINGTON.—Miss Maud Jones has been appointed Matron of the Cottage Hospital, Bridlington. Miss Jones received her training at the Burnley Victoria Hospital, where she has recently acted as night superintendent, and subsequently had charge of the women's ward.

WHALLEY, LANCASHIRE.—Miss Hilda Reece has been appointed District Nurse, of Whalley, Lancashire. Miss Reece received her training at the Victoria Hospital, Burnley.

ISOLATION HOSPITAL, BIGGLESWADE.—Miss Annie Bontel has been appointed Nurse-Matron of this hospital. She was trained at Lambeth Infirmary, and has since held the post of nurse-in-charge of the Isolation Hospital, Blaby.

### Minor Appointments.

POPLAR AND STEPNEY SICK ASYLUM DISTRICT.—Miss Jessie Isabella Smith was appointed Sister of the Poplar and Stepney Sick Asylum District on July 20th, 1897. She was trained at the Royal Infirmary, Aberdeen, where she subsequently held the post of staff nurse. She afterwards spent seven months in private work.

SOUTH METROPOLITAN DISTRICT SCHOOL, BANSTEAD ROAD.—Miss Alice Georgina Maple has been appointed Infirmary Nurse, on July 26th, of the Banstead Road Girls' School. She was trained at St. Olave's Infirmary, Rotherhithe, and has previously held a position as nurse at the above infirmary.

CITY ISOLATION HOSPITAL, NORWICH.—Miss Alice Bayne was appointed Charge Nurse on July 26th of the City Isolation Hospital, Norwich. She was trained at the North-Eastern Hospital (M.A.B.) and has held the position of first-class assistant nurse at the above hospital.

FOREST GATE DISTRICT SCHOOL INFIRMARY.—Miss Matilda Atkins was appointed Nurse of the above infirmary on July 15th. She was trained at St. Bartholomew's Hospital, the City of London Hospital, and at the Highgate Infirmary, and has since that time been engaged in private work.

CLATTERBRIDGE, CHESHIRE. — Miss Blake has been appointed Superintendent Fever Nurse at the Wirral Joint Hospital on July 23rd. She was trained at the Monsell Hospital, Manchester.

BROOK HOSPITAL.—Miss Blanch Cross was appointed on July 22nd First Assistant Nurse to the Brook Hospital. She was trained for two years at the Children's Hospital, Pendlebury.

JOHNSTONE AND DISTRICT COTTAGE HOSPITAL.—Miss E. Froggatt has been appointed Nurse of the Cottage Hospital. She was trained at Dewsbury and District General Infirmary.

### Notes and Queries.

The contents of the Editor's Letter-box have now reached such unwieldy proportions that it has become necessary to establish a hard and fast rule regarding Answers to Correspondents. In future, all questions requiring replies will continue to be answered in this column without any fee. If an answer is required by letter, a fee of half-a-crown must be enclosed with the note containing the enquiry. We are always pleased to help our numerous correspondents to the fullest extent, and we can trust them to sympathise in the overwhelming amount of writing which makes the new rules a necessity. Every communication must be accompanied by the writer's name and address, otherwise it will receive no attention.

*District Nursing and "Simple Ointment."*

(325) Kindly tell me of some book which contains all information required for district nursing. I am hospital trained, but find I require to know more of the usual applications for ulcered legs and usual chronic cases. I have the one belonging to the Burdett series, but find it hardly what I require. (2) Can you tell me what is meant by simple ointment, an expression I never heard in the hospital?—*Lavington.*

The question of proper treatment of ulcers must be referred to the medical attendant, as no book on nursing can give the necessary medical information. (2) "Simple" ointment is one composed chiefly of wax and lard, and is in common use by some practitioners.

*Nurses' Fees before Confinement.*

(326) Is it the rule to charge full fees for the weeks in the home before confinement? I have been three weeks?—*Nurse Mary.*

It is not quite clear what is meant here. A monthly nurse charges full fees from the day she enters her patient's house after being sent for.

*Fever Training.*

(327) I should be grateful if you could give me any information on the subject of fever hospitals. I am anxious to get some good fever training, but do not know where it would be best to apply. Can you recommend any hospital in particular? I have had three years' training in a children's hospital, and one year in general training here. I suppose I should be eligible for the post of charge nurse?—*Fever.*

The larger Metropolitan Asylums Board Hospitals give excellent training. Watch the advertisement columns of the "Local Government Board Journal" and THE HOSPITAL, and if that be insufficient, advertise in them.

*Nursing at Johannesburg.*

(328) Will you kindly give me all information you can as to how to obtain a post in a hospital or private nursing at Johannesburg.—*Sister.*

Write for information to Sister Adele, lady superintendent of the nursing staff, at the hospital, Johannesburg, Transvaal. We draw "Sister's" attention to the fact that nurses have very great difficulties to contend with in South Africa, and that she must be careful. If she obtain work, the United British Women's Emigration Association, Imperial Institute, S.W., would advise her as to the best and safest way of reaching her destination. This association gives facilities of passage to suitable women emigrants not available in the usual course.

*The Newest Medical Dictionary.*

(329) Would you tell me the name of the newest dictionary and the price, as there are several terms used now that were not when I was trained?—*Sister Gertrude.*

The Scientific Press, 28 & 29, Southampton Street, Strand, publish a handy little volume at 2s., by Honnor Morten, entitled "The Nurses' Dictionary of Medical Terms and Nursing Treatment."

*Nursing at the Cape.*

(330) Will you kindly tell me to whom to apply respecting nursing at the Cape under the Victoria Nursing Institute?—*Kate Gossage.*

We are informed by Miss Peter that the Victoria Nursing Institute, Cape Town, is not affiliated with the Queen Victoria Jubilee Nursing Institute. We know nothing of it.

*A Weir-Mitchell Hospital.*

(331) Will you kindly tell me if there is a home or hospital in England corresponding to the Weir-Mitchell Hospitals, and what are the lowest fees?—*B. M. Grimbley.*

There are no hospitals conducted on the Weir-Mitchell system in England, but the treatment is carried out by some private medical men.

*The Higher Post.*

(332) Will you kindly tell me which is the higher post. That of charge nurse to the body of the infirmary or to the blocks; (2) Will you kindly tell me what are your charges?—*E. S. Dixon.*

There can be no difference in position; it is simply a matter of work. Both nurses in the case you mention would be charge nurses and rank equally. (2) We charge nothing for replying in this column, but a fee of 2s. 6d. is necessary if a private answer be required.

### Wants and Workers.

THE attention of correspondents is directed to the fact that "Helps in Sickness and to Health" (Scientific Press, 28 & 29, Southampton Street, Strand, London, W.C.) will enable them promptly to find the most suitable accommodation for difficult or special cases.]

BED REST.—Will the nurse who asked for a bed rest kindly send her address, as one has been offered for the use of her patient, and her address has beenaid. misl

